# Effectiveness and Safety of Endoscopic Treatment of Benign Biliary Strictures Using a New Fully Covered Self Expandable Metal Stent

**DOI:** 10.1155/2013/183513

**Published:** 2013-05-11

**Authors:** Mihir S. Wagh, Disaya Chavalitdhamrong, Koorosh Moezardalan, Shailendra S. Chauhan, Anand R. Gupte, Michael J. Nosler, Chris E. Forsmark, Peter V. Draganov

**Affiliations:** Division of Gastroenterology, Hepatology and Nutrition, University of Florida, 1600 SW Archer Road, Room HD 602, P.O. Box 100214, Gainesville, FL 32610, USA

## Abstract

*Background*. In patients with benign biliary strictures, the use of fully covered self-expandable metal stents (SEMS) has been proposed as an alternative to plastic stenting, but high quality prospective data are sparse. This study was performed to evaluate the long-term effectiveness and safety of a new fully covered SEMS for benign biliary strictures. *Methods*. All consecutive patients with benign biliary strictures were treated with placement of a fully covered SEMS (WallFlex) for 6 months. Short- and long-term stricture resolution, adverse events, and ease of stent removal were recorded. *Results*. 23 patients were enrolled. Stricture etiology was chronic pancreatitis (14), postorthotopic liver transplant (4), idiopathic (4), and biliary stones (1). All ERCPs were technically successful. All stents were successfully removed. Short-term stricture resolution was seen in 22/23 (96%) patients. Long-term success was 15/18 (83.3%). All 3 failures were patients with biliary strictures in the setting of chronic calcific pancreatitis. *Conclusions*. The use of the new SEMS for the treatment of benign biliary strictures led to short-term stricture resolution in the vast majority of patients. Over a long-term followup the success rate appears favorable compared to historical results achieved with multiple plastic stenting, particularly in patients with chronic pancreatitis. The study was registered with ClinicalTrials.gov (NCT01238900).

## 1. Introduction

Benign biliary strictures are common and challenging clinical problems. The two leading etiologies are chronic pancreatitis (CP) and postoperative complications, either related to bile duct injury at the time of cholecystectomy or anastomotic narrowing after orthotopic liver transplantation (OLT). The reported incidence of benign biliary strictures among CP patients ranges from 3 to 46% [1]. In post-OLT setting, biliary tract complications can occur in up to 30% of patients [2]. Furthermore, biliary strictures can occur in 0.5 to 0.9% of patients undergoing laparoscopic cholecystectomy [3, 4]. Other less frequent causes are primary sclerosing cholangitis, papillary stenosis, autoimmune pancreatitis, and bile duct stones [5]. Benign biliary strictures can present with a variety of clinical scenarios that range from mild elevation of liver enzymes to recurrent episodes of cholangitis to secondary biliary cirrhosis and end stage liver disease [6]. In order to prevent these serious complications, proper and early effective treatment of these strictures is essential.

Traditionally, benign biliary strictures had been treated surgically. Currently, endoscopic retrograde cholangiopancreatography (ERCP) with placement of multiple plastic stents has become the first line therapy. In the setting of postoperative biliary strictures, ERCP has shown a high rate of stricture resolution ranging from 75% to 89% [7–9]. The main drawbacks of the ERCP-based approach are the need for multiple procedures and a treatment period that usually lasts over a year. Furthermore, relatively high morbidity can occur due to stent occlusion [10], and endoscopic therapy for CP-related biliary stenosis has reported success rates of approximately only 25% [11]. 

In order to decrease the incidence of cholangitis and the need for multiple ERCPs, uncovered or partially covered self-expandable metal stents (SEMS) have been used. However, these stents have high rates of stent clogging caused by tissue ingrowth which could cause challenges with stent removal [10, 12]. Fully covered SEMS have been used with more favorable results; however, stent migration is a main concern [13, 14]. Only limited high quality data are available on the long-term efficacy and safety of fully covered SEMS in the treatment of benign biliary strictures [13, 15–22]. Therefore, we prospectively investigated the short- and long-term effectiveness and safety of a new fully covered SEMS in patients with benign biliary strictures.

## 2. Materials and Methods

This was a prospective cohort study. The study was approved by the Institutional Review Board (IRB) at the University of Florida. All patients signed informed consent. The study concept, hypothesis, and design were investigator initiated, and no financial support or free devices were received. The aim of this study was to evaluate the long-term effectiveness and safety of a new fully covered SEMS for benign biliary strictures.

### 2.1. Patients

Consecutive patients referred to the University of Florida for ERCP for benign biliary strictures were considered for study enrollment from July 2009 to December 2011. The inclusion criteria were strictures located at a minimum 2 cm downstream to the bifurcation and due to one of the following etiologies: CP, post-OLT, postcholecystectomy, postradiation or chemotherapy (for nonbiliary malignancies), bile duct stone related strictures, and idiopathic. Patients with prior endoscopic attempts with dilation and plastic stents were also included. The exclusion criteria were patients with bile duct strictures less than 2 cm from the bifurcation, autoimmune pancreatitis, primary sclerosing cholangitis, malignant strictures, age less than 18, and pregnancy.

### 2.2. Endoscopy Protocol

**Figure 1:**
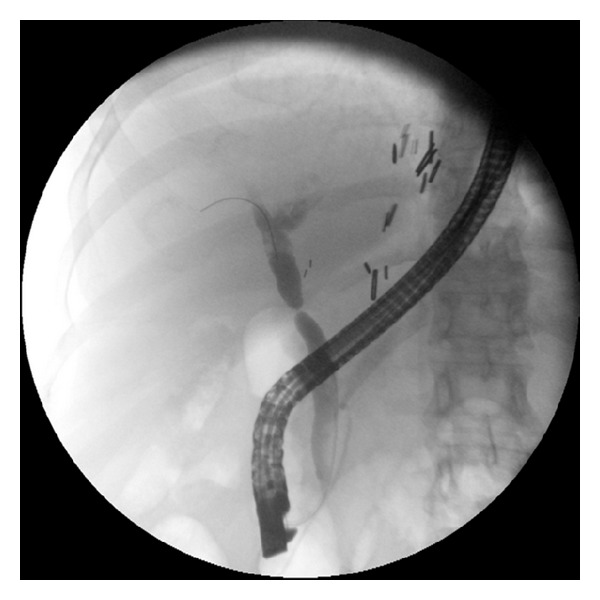
Cholangiogram of a patient with postliver transplant anastomotic stricture.

**Figure 2:**
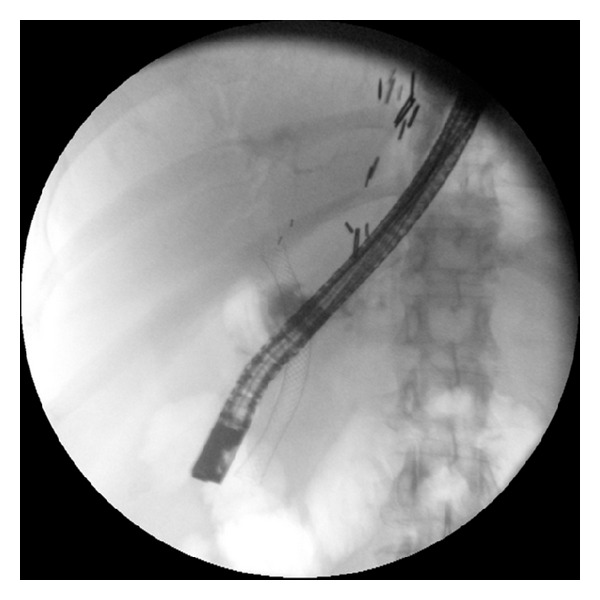
Fully expanded metal stent bridging the stricture.

**Figure 3:**
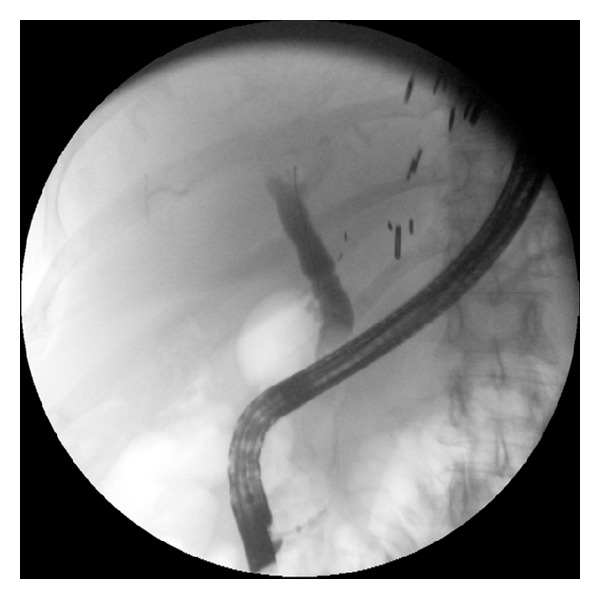
Resolution of the stricture.

ERCP was performed by one of our four experienced therapeutic endoscopists (MSW, SSC, CEF, and PVD) using the TJF 160V duodenoscope (Olympus America, Center Valley, PA, USA). The stents used in this study were the fully covered WallFlex Biliary RX Stents (Boston Scientific Corporation, Natick, MA, USA) which are available in diameters of 8 or 10 mm and lengths of 40, 60, and 80 mm. Biliary sphincterotomy, if not done previously, was performed. Stricture dilation was not preformed prior to stent insertion. All stents were placed with the distal end extending into the duodenum. Per protocol, the intent was to keep the stent in place for 6-months. If the stricture had resolved at the 6-month follow-up ERCP, patients were classified as short-term success and then entered in the follow-up portion of the study. If at the time of the 6 month stent removal, the stricture was improved but still present, then a new fully covered SEMS (WallFlex) was placed. If the stricture had resolved at the time of the second stent removal, the patient was also classified as “short-term success.” If the stricture was still present, the patient was classified as “short-term failure.” A typical stenting sequence is shown on Figures 1, 2, and 3. Stents were removed by grasping the dedicated retrieval loop at the proximal end of the stent using a rat-tooth forceps. If this was not feasible, the distal end of the stent was captured with a snare and then retrieved. Patients with post-OLT anastomotic biliary strictures received antibiotic prophylaxis prior to and up to 3 days after the stent placement to prevent cholangitis.

### 2.3. Outcomes

The primary outcome of this study was short- and long-term success rate in resolution of biliary strictures. Secondary outcomes were the frequency and severity of adverse events (including stent migration), duration and number of endoscopic treatment(s), and ease of stent removal.

### 2.4. Definitions

The procedure was considered technically successful if all of the following criteria were met: achieving deep bile duct cannulation, traversing the stricture with a wire, and deploying the SEMS across the stricture. For the follow-up ERCP, the procedure was considered technically successful if the SEMS could be extracted. The ease of stent extraction was graded on a 4-point scale (with ease, mild difficulty, significant difficulty, and failed). Short-term success was defined as resolution of the stricture as documented by rapid drainage of contrast out of the proximal biliary tree and easy passage of stone extraction balloon inflated to the size of the proximal bile duct. Long-term success was defined as no clinical evidence of recurrence of the biliary stricture during the follow-up period as documented by laboratory findings or imaging and no further need for further endoscopic or surgical interventions. The long-term follow-up was at least a 12-month period. The followup period started at the time of the metal stent removal. Adverse events were defined and graded using the 2010 American Society for Gastrointestinal Endoscopy consensus criteria [23]. 

### 2.5. Statistical Analysis

IBM SPSS version 19 was used for statistical analysis. The level of significance was set at *P* < 0.05 in statistical analyses. Per-protocol and intent-to-treat analysis was carried out. 

## 3. Results

A total of 23 patients (10 male; mean age: 60.6; range: 24–81 years) were enrolled. Stricture etiologies were CP (14), OLT (4) (mean time after OLT was 2.7 months), idiopathic (4), and history of biliary stones (1). Patient characteristics are summarized in Table 1. 

**Figure 4:**
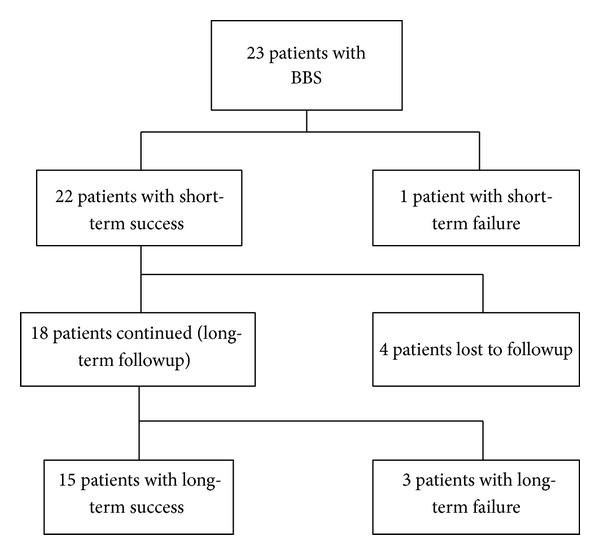
Flow diagram of the patients through the study.

Of the 23 patients that completed the endoscopy protocol, 22 patients entered the followup phase (one patient had a persistent stricture and was therefore classified as short-term failure). Long-term follow-up was obtained in 18/22 (81.8%) of the patients. Therefore, 23 patients were available for the analysis of the technical success of the ERCPs and the short-term success rate of stricture resolution, and 18 patients were available for the analysis of the long-term success rate of stent therapy. Patient flow through the study is summarized on Figure 4.

All ERCP procedures were technically successful in all patients. The average number of ERCPs performed per patient was 2.4 (range 2–4). The average total stenting period per patient was 7.5 months (22 days to 17.6 months). Stent migration was seen in 9/23 (39.1%) patients (5 downstream, 4 upstream). In the cases of stent migration, all of the stent was still bridging the stricture except in two cases. In the first patient, the metal stent migrated out of the bile duct completely; however, the biliary stricture had resolved with no recurrence during followup. The second case was a patient with post-OLT anastomotic stricture at the upper third of the common bile duct. The stricture was located 70 mm proximal to the ampulla, and on the first ERCP a decision was made to deploy the longest size SEMS that was available (80 mm). The proximal end of the stent at the end of the deployment was 10 mm proximal to the stricture. On the followup ERCP, the proximal end of the stent was found below the stricture. Since stents longer than 80 mm are not manufactured, a decision was made to switch to plastic stents, and the patient was classified to be a short-term failure. 

All stents were successfully removed in all patients. Stent removals were graded as “easy” except in 2 cases (8%) with upstream stent migration where the removal was graded as “with significant difficulty.” Associated pathologies included 7 patients with common bile duct stones. Balloon extraction of the stones was successful in all cases. 

The overall short-term treatment success rate for stricture resolution at the end of the stenting period was seen in 22/23 (95.6%) patients. The short-term success rate was 14/14 (100%) among the CP patients, 3/4 (75%) among the OLT patients, and 5/5 (100%) in remaining patients. The short-term failure was a patient with post-OLT anastomotic stricture as discussed in detail in the previous paragraph.

Long-term followup was available in 18/22 patients (81.8%). Patients were followed for a median follow-up period of 18.8 months (interquartile range of 14.1 to 21.3 months). The overall long-term success rate of stricture resolution was 15/18 (83.3%): in the CP group 8/11 (72.7%), in the OLT group 3/3 (100%), and the remaining patients 4/4 (100%) (Table 2). The failures included three patients with distal biliary strictures in the setting of CP with calcification. One patient underwent surgery with hepaticojejunostomy, and two patients needed further endoscopies with plastic stents.

The overall long-term treatment success on an intention to treat basis was 15/22 (68.2%): in the CP group 8/12 (66.7%), in the OLT group 3/3 (100%), and in the remaining patients 4/7 (57.1%) (Table 2). This analysis considered all patients lost to followup as long-term therapeutic failures, in a “worst-case” scenario analysis. 

Adverse events included severe pain in one patient after placement of 10 mm wide stent. The stent had to be removed due to the pain; however, the patient subsequently did well with an 8 mm diameter stent, and the stricture was successfully treated with no recurrence during long-term followup. There was no incidence of post-ERCP pancreatitis or cholangitis. No other adverse events were noticed during followup period. Of note, in all 13 (56.5%) patients with gallbladder in situ, the SEMS was placed across the takeoff the cystic duct, and no acute cholecystitis was observed.

## 4. Discussion

Endoscopic treatment of benign biliary strictures has gained popularity as a less invasive approach compared to surgery. The preferred endoscopic strategy has been to sequentially dilate the stricture and insert incremental number of plastic stents during multiple consecutive endoscopic sessions [24–27]. Although good outcomes have been reported in patients with postoperative biliary strictures, the success rates in the setting of chronic pancreatitis have been disappointingly low [27, 28]. Furthermore, long-term success with plastic stents requires multiple endoscopic procedures which increase risks, costs, and patient burden [29]. 

Metal stents have been used in an attempt to improve the long-term success rate. The use of uncovered and partially covered SEMSs was abandoned due to the very high rate of tissue ingrowth, which leads to clogging of the stent and significant difficulty in stent removal [10, 12]. Recently, the use of various types of fully-covered metals has gained popularity, although concerns remain regarding safety and long-term outcomes [13, 16–22]. Our study expands on previously reported experiences with various fully covered SEMS and is the first one to report the prospective long-term outcomes with the use of the WallFlex Biliary RX Stent for benign biliary strictures. Summarized data from prior studies on other fully covered SEMS for benign biliary strictures with long-term followup are shown in Table 3.

Our data revealed that in patients with benign biliary strictures, placement and removal of fully-covered SEMS are technically highly successful (100%) and led to short-term stricture resolution in the vast majority of the patients (96%). Furthermore, long-term stricture resolution was achieved in 83% of the patients. Moreover, our long-term success rate in patients with CP-related strictures appeared significantly higher (73%) compared with prior experiences with plastic stents and other types of SEMS [13, 16]. Importantly, these successful outcomes were achieved with a very low number of ERCPs (mean of 2.4 procedures per patient). The vast majority of patients required only one ERCP for stricture resolution by SEMS placement (due to its radial expansion capability) and one ERCP for stent removal. This suggested the benefit of SEMS over plastic stents to overcome the problem of multiple plastic stent placements with repeated interventions.

 We observed an overall very low incidence of adverse events. We noted only one episode of postprocedure pain due to continuous radial expansion force of the SEMS, and that was successfully treated by downgrading the stent size to a smaller diameter. There were no complications during stent placement or stent removal, and no stent dysfunction was noted. As we expected, stent migration was common (9/23; 39%), but it was without clinical consequences except in one case. All stents were successfully removed with ease. This could be explained by having loop for stent retrieval. The only two cases with significantly difficult removals occurred with upstream stent migration. After we encountered this problem with upstream migration, we modified our technique to leave 4 to 5 mm of the distal end of the stent protruding from the ampulla at the end of stent deployment.

We believe that our study provides preliminary evidence of the long-term clinical utility of fully-covered SEMS as a therapy for benign biliary strictures. Our study has several methodological strengths: (1) prospective design; (2) strict and clinically meaningful predetermined definitions for procedure, short-term and long-term success; (3) predetermined treatment protocol; (4) included all patients presenting to our center which led to reducing the potential for selection bias; and (5) relatively long clinical follow-up period (mean of 18 months).

We also acknowledge the following limitations of this study: (1) relatively small number of patients. As a result, our study lacks the power to provide a meaningful comparison between various subgroups. (2) The patients included in this study were referred to a tertiary referral center. This reason raises the concerns about selection bias of more difficult cases. (3) Fifty-six percent of our patients had prior therapy for their benign biliary stricture with dilation and plastic stent placements; therefore they were not treatment naive. (4) We had a high prevalence of CP-related strictures which are known to be more difficult to treat. (5) Four patients were lost to followup. Although our “loss to followup” rate is within the methodologically expectable rate, we unfortunately could not obtain long-term followup in all patients. In order to account for this, we performed an intention-to-treat analysis which we considered all of the patients that were lost to followup to be failures of therapy. Therefore, this analysis should provide a “worst case scenario” interpretation of our data.

Overall, we achieved very good results, and the biliary stricture resolution rate in CP cases appeared to be superior to historical results achieved with plastic stenting. Furthermore, recurrence of the stricture in three patients happened in CP patients. This might be explained by the nature of the disease itself, not from the de novo stricture caused by SEMS. However, this should be interpreted with caution with the absence of a control group. 

## 5. Conclusions

The use of fully-covered SEMSs for the treatment of benign biliary strictures leads to short-term stricture resolution in the vast majority of patients. Over a long-term follow-up period, the success rate appears favorable compared with historical results achieved with multiple plastic stenting, particularly in the patients with CP related strictures. Future randomized studies directly comparing plastic versus metal stents appear warranted. An optimal quality of fully-covered SEMS needs to be developed in order to make this stent a standard of care tool for patients suffering from benign biliary strictures with the best outcome and most cost effectiveness.

## Figures and Tables

**Table 1:** Patient characteristics.

Number	23
Age (years)	60 (51–70)
Male (% men)	10 (43%)
Etiology	
Chronic pancreatitis	14 (61%)
Orthotopic liver transplantation	4 (17%)
Idiopathic	4 (17%)
Gallstone related	1 (4%)
Prior common bile duct stenting	13 (56%)

Values listed as number (%) or median (IQR).

**Table 2:** Outcomes.

	Overall	Chronic pancreatitis	All others	*P* value
Short-term success	22/23 (96%)	14/14 (100%)	8/9 (89%)	0.39
Long-term success				
Per-protocol analysis	15/18 (83%)	8/11 (73%)	7/7 (100%)	0.21
Intention-to-treat analysis	15/22 (68%)	8/12 (67%)	7/10 (70%)	0.65

**Table 3:** Prior prospective studies on fully covered SEMS for benign biliary strictures.

Study (year)	*N *	Type of stent	Stricture resolution success rate	Migration rate	Mean followup (months)
Traina et al. (2009) [17]	16	Niti-S Comvi	87.5%	38%	10.1
Mahajan et al. (2009) [13]	44	GORE VIABIL	83%	4.5%	3.8
Hu et al. (2011) [18]	13	A short stent with a retrieval suture	92.3%	None	12.1
Park et al. (2011) [19]	22	SEMS with anchoring flap	91%	None	3.6
	21	SEMS with flared end	88%	33%	4.2
Tarantino et al. (2012) [20]	39	Niti-S Comvi (after failure of conventional therapy)	71.8%	33.3%	22.1
	15	Niti-S Comvi (as first approach)	53.3%	46.7%	14.4
Poley et al. (2012) [16]	23	HANAROSTENT	61%	4%	15
Perri et al. (2012) [21]	7	Unflared end Niti-S	43%	100%	24
	10	Flared end Niti-S	90%	40%	24
Tarantino et al. (2012) [22]	62	Niti-S Comvi	90.3%	24.2%	15.9
Current study	23	Wallflex	96%	39.1%	18.8
